# Global Access to Essential Medicines for Childhood Cancer: A Cross-Sectional Survey

**DOI:** 10.1200/JGO.18.00150

**Published:** 2018-11-30

**Authors:** Phillip Cohen, Paola Friedrich, Catherine Lam, Sima Jeha, Monika L. Metzger, Ibraham Qaddoumi, Paula Naidu, Lane Faughnan, Carlos Rodriguez-Galindo, Nickhill Bhakta

**Affiliations:** **Phillip Cohen**, Children’s Hospital of Philadelphia, Philadelphia, PA; **Phillip Cohen**, Centre for Global Health, Trinity College Dublin, Dublin, Ireland; and **Paola Friedrich**, **Catherine Lam**, **Sima Jeha**, **Monika L. Metzger**, **Ibraham Qaddoumi**, **Paula Naidu**, **Lane Faughnan**, **Carlos Rodriguez-Galindo**, and **Nickhill Bhakta**, St Jude Children’s Research Hospital, Memphis, TN.

## Abstract

**Purpose:**

Global data mapping access to essential chemotherapeutics for pediatric cancer are scarce. We report a survey of international pediatric cancer care providers’ access to these medicines.

**Methods:**

A Web-based survey was sent to pediatric oncologists registered on the Cure4Kids Web portal. We queried chemotherapeutics in the WHO Essential Medicines List for Children, from which the average proportional availability was summarized as each country’s access score. In addition, we examined availability of drug packages defined by the WHO-sanctioned Expert Committee for eight pediatric cancers. We undertook a sensitivity analysis investigating how regimen access would change if the cytotoxics specified in recent agreements between the Clinton Health Access Initiative, American Cancer Society, and pharmaceutical companies were universally available.

**Results:**

There were significant (*P* < .001) differences in the median access scores between World Bank income groups, and 42.9% of respondents from low-income and lower middle–income countries reported suboptimal access scores. Our disease-based analysis revealed that 42.1% of patients in low-income and lower middle–income countries lacked full access to chemotherapy packages. Guaranteed availability of the cytotoxics specified in the Clinton Health Access Initiative/American Cancer Society agreements was projected to increase this regimen-based access by 1.6%, although including four additional chemotherapeutics would further increase coverage by 13.9%.

**Conclusion:**

This study is the first, to our knowledge, to assess worldwide variation in practical access to pediatric chemotherapy. Although mapping the proportion of available chemotherapeutics is informative, we also developed a meaningful estimate of access using disease-specific drug packages. These data provide an important baseline for continued monitoring and can aid in planning adaptive treatment guidelines that consider the trade-offs between access and outcomes.

## INTRODUCTION

Incremental improvement of childhood cancer survival is both a landmark achievement of modern medicine and a sobering reminder of the vast inequities that exist for children globally. Although cure rates in high-income countries (HICs) now exceed 80%, more than 90% of children with cancer are diagnosed in low- and middle-income countries (LMICs), where survival is far lower.^1^ In recognition of this gap, in May 2017, the WHO passed the global cancer resolution WHA70.12, which includes childhood cancer in its cancer control mandate.^2^ Understanding and addressing global variations in treatment access will be required to inform its implementation.

Despite an increasing profile in the Sustainable Development Goals, measuring access to essential medicines has proven notoriously difficult and represents the only Millennium Development Goal target (8E) removed from annual progress reports.^3,4^ Data describing access to pediatric chemotherapeutics are particularly lacking, with only one published survey of national formularies available.^5^ Because formularies represent a problematic proxy for clinical stocks, a recent statement from the International Society of Pediatric Oncology emphasized the need for more data on access to chemotherapeutics in the WHO Essential Medicines List for Children (EMLc).^6^ These data are important considering the recent landmark agreements between the Clinton Health Access Initiative (CHAI), the American Cancer Society (ACS), and multiple pharmaceutical companies to facilitate procurement of selected chemotherapy drugs in six low-income countries (LICs) and lower middle–income countries (L-MICs).^7^ Without knowing which pediatric-specific cytotoxics are truly available where, actors such as the CHAI and ACS are left negotiating blind. To provide a comprehensive set of baseline data and propose a clear methodology for future efforts, we aimed to map the practical availability of EMLc chemotherapeutics around the world for the first time.

## METHODS

### Study Population

A cross-sectional quantitative survey was sent to pediatric oncologists on Cure4Kids (www.cure4kids.org), a free online portal developed at St Jude Children’s Research Hospital (SJCRH) for those in pediatric cancer care. Cure4Kids has been previously used to host research surveys on pediatric cancer across geography and World Bank income categories.^8-10^

Physicians were the primary target of the e-mail invitation but were permitted to delegate the survey to another health care or research professional. Inclusion and exclusion criteria were defined to make the conclusions as generalizable as possible for most patients around the world. Only respondents who worked at treatment centers that accepted public patients were eligible. Respondents from centers that specialized in other fields and/or only treated a small subset of cancers were excluded. Finally, participants who answered less than 80% of drug availability questions or responded that less than 80% of drugs were necessary to treat cancer at their centers were excluded.

### Survey Design and Distribution

The survey tool (Data Supplement) was newly developed by the authors with the WHO-Health Action International (WHO-HAI) methodology used as a reference, which sets a benchmark of 80% to define optimal availability. The WHO-HAI methodology is generally used to monitor a core list of medications in primary care settings within a single country, and a previous review did not identify studies using this approach to monitor chemotherapeutics.^11^

A total of 29 medications were surveyed, on the basis of work from the Expert Committee sanctioned by the WHO to provide recommendations for the EMLc (Data Supplement).^12,13^ Among these drugs were the 22 listed in the 2017 EMLc’s “Cytotoxics and Adjuvants” section, which accounted for the medicines formulating each respondent’s access score (Table 1).^14^ The access score was defined by the proportion of these 22 drugs deemed routinely available to all appropriate patients. Although the EMLc also includes four corticosteroids cited in the Expert Committee’s recommendations, it includes them in a separate subsection.^15^ Given this distinct categorization, they were not included in the access score calculation.

**Table 1:**
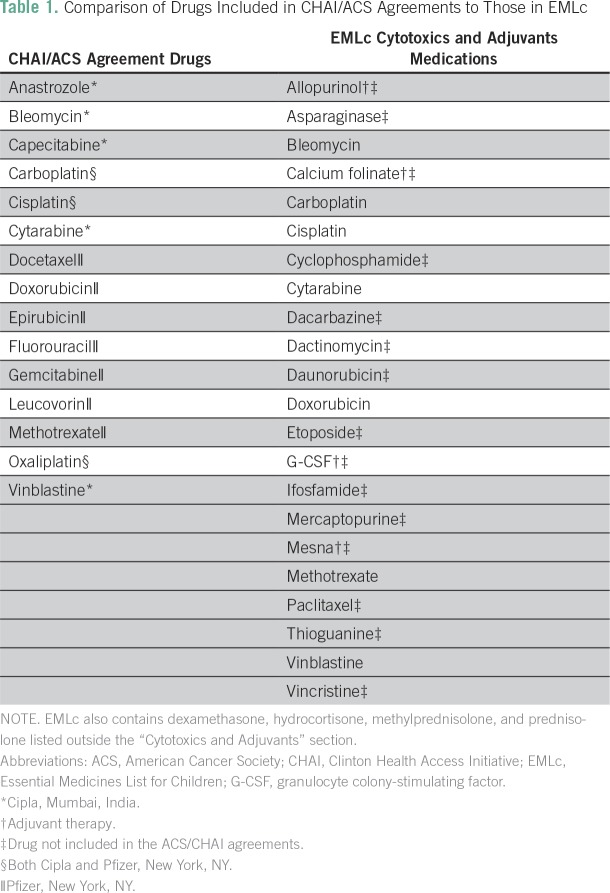
Comparison of Drugs Included in CHAI/ACS Agreements to Those in EMLc

Institutional review board approval was granted by SJCRH and Trinity College Dublin. The survey was e-mailed on May 8, 2017, to the 2,590 Cure4Kids users registered as pediatric hematologists or oncologists who had accessed the site since January 2016. Twenty-seven program directors working at SJCRH partner sites internationally were also included. E-mail reminders were sent weekly to those who failed to respond before the survey was closed after 1 month. The survey was written in English using SurveyMonkey, and study consent was obtained electronically before respondents began the survey.

### Statistical Analysis

Survey responses were manually reviewed and then anonymized. Although multiple responses from a single institution were accepted and weighted equally, duplicate entries from the same e-mail address were removed with the first entry retained. Responses were grouped by country to calculate mean access scores and coded into four ordinal groups. Countries were categorized as having ideal (95% or greater proportional availability), optimal (80% or greater), suboptimal (60% or greater), or poor (less than 60%) mean access scores. Because the distribution was skewed, nonparametric statistical tests (Kruskal-Wallis with Bonferroni adjustments, Mann-Whitney *U*) were used to test for significance (threshold of *P* ≤ .05). These data were also stratified by population age 0 to 14 years within each World Bank income group (fiscal year 2017 to 2018) to describe the population share subject to graded levels of drug access.^15^

We also conducted a sensitivity analysis to investigate access to disease-specific treatments by applying a regimen-based approach for eight pediatric cancers where consensus-adapted regimens have been proposed (Data Supplement). Seven of these regimens were described by the WHO-sanctioned Expert Committee making EMLc recommendations.^12^ The exception was for acute lymphoblastic leukemia. Here, the simplest regimen for resource-limited settings described by Hunger et al^16^ was used because it includes only six drugs (similar in quantity to the other included regimens) and has been considered a bare minimum treatment regimen in LMICs.^12^ Because effectiveness has been typically demonstrated with complete regimens, routine access to all drugs in an adapted regimen was necessary to infer local access to treatment. All sensitivity analyses used absolute incidence numbers of new diagnoses from GLOBOCAN 2012 and relative proportion of childhood cancers from the SEER Program to estimate the total number of patients with the eight cancers within World Bank income groups.^17,18^ This allowed us to analyze regimen-based access using the relative frequency of each cancer and apply results to global patients.

Because the sample pool among LICs was small, respondents from LICs were combined with those from L-MICs (Data Supplement). First, we calculated a baseline of coverage of each cancer for each income group. Then, we calculated the increase in coverage contingent upon guaranteed worldwide availability of medications in the CHAI/ACS agreements, without and with the addition of four high-impact EMLc chemotherapeutics not included in the current CHAI/ACS agreements. Respondents within World Bank income groups were not stratified by country or population share in these analyses.

Before addressing the study objectives, internal validity was investigated. We calculated Cronbach’s α (α = .885) for drugs making up the access scores. We also tested responses to the question, “Does lack of chemotherapy hinder treatment at your center?” against access scores. In both cases, the data showed high internal consistency (Data Supplement).

## RESULTS

**Fig 1:**
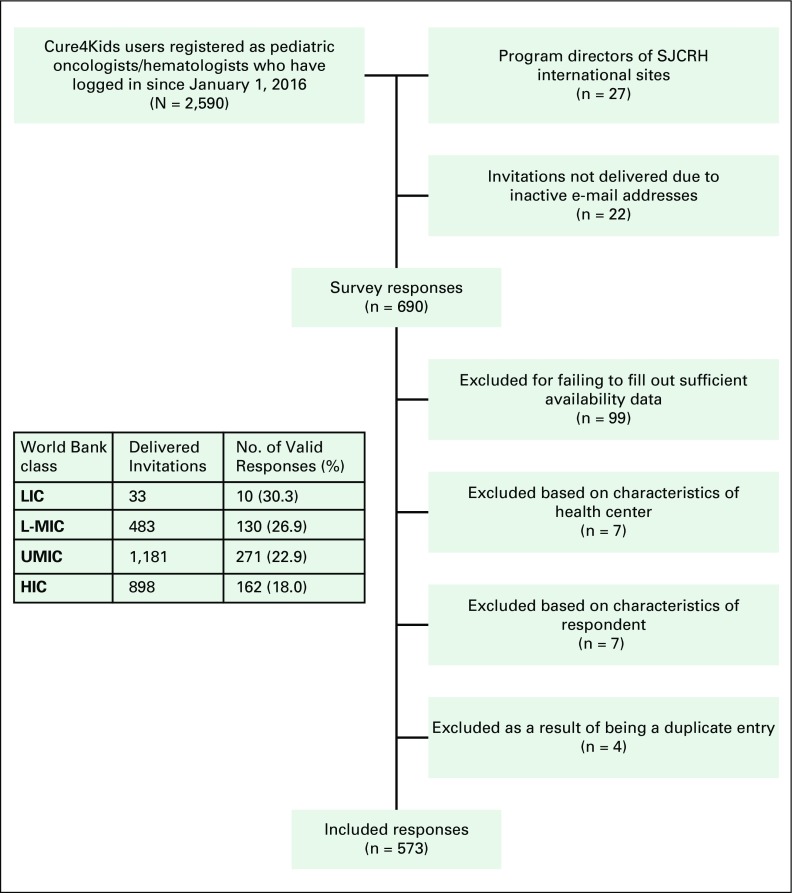
Flow diagram describing recruitment and evaluation of study participants. HIC, high-income country; LIC, low-income country; L-MIC, lower middle–income country; SJCRH, St Jude Children’s Research Hospital; UMIC, upper middle–income country.

Figure 1 and Table 2 describe the study participants’ selection process and demographics. Of the 2,595 delivered invitations, there were 690 responses (26.6%), and 573 respondents (22.1%) were included (Fig 1). The response rates were significantly higher among participants in LICs and L-MICs, with participants from HICs being least likely to respond (*P* = .001). Nonetheless, the fewest total responses were from LICs. The survey was sent to participants from 137 countries, and responses from 94 countries were included in the final analysis, with a relative under-representation of African, Southeast Asian, and Western Pacific responders compared with their share of the world’s age 0 to 14 population.^19^ In all groups, government was the foremost payer of chemotherapy, but among LIC/L-MIC respondents, nongovernmental organizations (21.3%) and out-of-pocket payments by families (21.3%) were also frequently cited (Table 2).

**Table 2:**
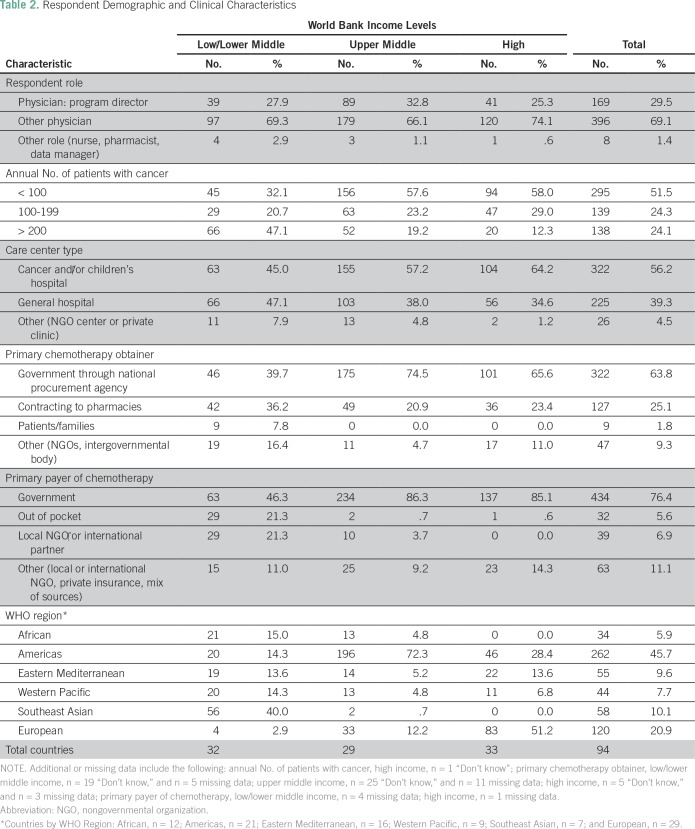
Respondent Demographic and Clinical Characteristics

**Fig 2:**
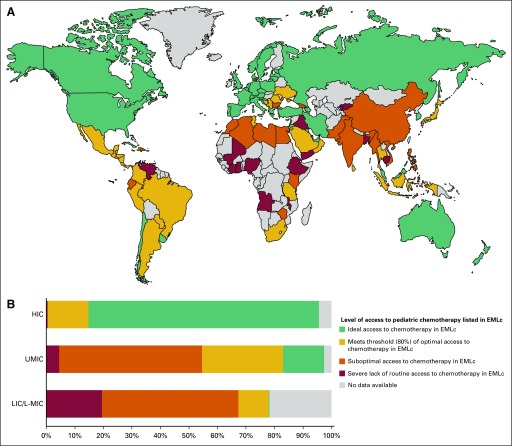
Global access to Essential Medicines List for Children (EMLc) cytotoxics and adjuvant medicines. (A) World map of countries’ average access scores. (B) Current global access to EMLc cytotoxics and adjuvant medicine relative to the population age 0 to 14 years. This figure is based on each country’s mean access score but weighs each country’s proportional share on the basis of its population age 0 to 14 years relative to other countries within its World Bank income group. Access score groupings are as follows: ideal access (greater than 95%; score of 20.9 to 22), optimal access (greater than 80%; score of 17.6 to 20.8), suboptimal access (greater than 60%; score of 13.2 to 17.5), and poor access (less than 60%; score of 0 to 13.1). No data available indicates no responses from country. HIC, high-income country; LIC, low-income country; L-MIC, lower middle–income country; UMIC, upper middle–income country.

When mapping access, HICs made up 28 of 37 countries with ideal access (28 of 33 of all HICs). No LIC had ideal access, and only two L-MICs had ideal access (Fig 2A). Of the additional 28 countries that met the WHO-HAI threshold for optimal access, half (14 of 28 countries) were upper middle–income countries (UMICs; 14 of 29 of all UMICs). A total of 29 countries had average access scores that were below the WHO-HAI threshold of optimal access, 20 of which were LICs/L-MICs (20 of 32 of LICs/L-MICs; Data Supplement).

We found statistically significant differences in access scores (*P* < .001; highest possible score, 22) between respondents from HICs (median score, 22; interquartile range [IQR], 21 to 22), UMICs (median score, 20; IQR, 18 to 22), and LICs/L-MICs (median score, 18; IQR, 12 to 21; *P* < .001 for each pair of groups). Overall, 42.9% of LIC/L-MIC respondents had suboptimal access scores (score of less than 17.6), compared with 24.0% of those from UMICs and 2.5% of those from HICs (*P* < .001). To quantify the impact of these results, we stratified national mean access scores by each country’s proportional share within their World Bank income group’s age 0 to 14 population. This stratification showed that these 94 countries include 86.9% of children age 0 to 14 years worldwide, including 95.5% of those in HICs, 97.4% in UMICs, and 78.3% in LICs/L-MICs (Fig 2B). Without accounting for countries outside the study, this analysis determined that 54.6% of youth in UMICs and 67.3% in LICs/L-MICs live in countries with suboptimal availability compared with 0.4% of youth in HICs (Fig 2B).

**Fig 3:**
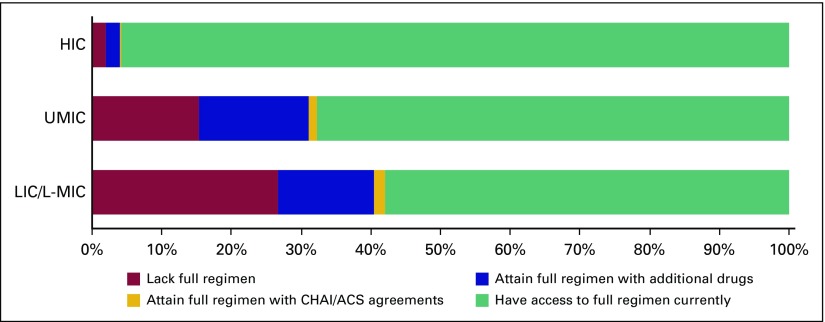
Relative incidence with access to full chemotherapy regimens for selected cancers. "Have access to full regimen currently" indicates proportion of patients across eight cancers that currently have routine access to all drugs in regimen. "Attain full regimen with Clinton Health Access Initiative (CHAI)/American Cancer Society (ACS) agreements" indicates proportion of patients who lack routine access to all regimen drugs currently but would attain full access with CHAI/ACS agreement drugs. "Attain full regimen with additional drugs" indicates proportion of patients who lack routine access to all regimen drugs currently but would attain full access with addition of CHAI/ACS agreement drugs and asparaginase, mercaptopurine, dactinomycin, and dacarbazine. "Lack full regimen" indicates proportion of patients who lack routine access to all drugs in regimen even with inclusion of CHAI/ACS agreement drugs and additional drugs. HIC, high-income country; LIC, low-income country; L-MIC, lower middle–income country; UMIC, upper middle–income country.

We next calculated regimen-based access in each World Bank income group across eight cancers. Per SEER estimates, the included cancers make up 47.5% of the overall childhood cancer burden (Data Supplement). Accounting for each included cancer’s epidemiology, our study found that 42.1% of applicable patients in LICs/L-MICs do not have access to the full disease-specific regimens (Fig 3). Superimposed on these data, we calculated the proportion of patients who would gain complete regimen access if guaranteed supply of the drugs included in the CHAI/ACS agreements (Table 3). For six of the eight cancers surveyed, less than 2% of LICs/L-MICs would achieve full access if guaranteed availability to the CHAI/ACS drugs was realized (Data Supplement). Across all income groups, our results indicated that global access to CHAI/ACS drugs would increase the percentage of patients with full regimen access by 1.2% (1.6% in LICs/L-MICs; Table 3). This minor impact reflects that although many regimens had at least one drug included in the CHAI/ACS agreements, the least available medications were not included. For example, the agreements included methotrexate (lacked by 18.6% of respondents from LICs/L-MICs) but not asparaginase (32.1%) or mercaptopurine (29.3%; Data Supplement).

**Table 3:**
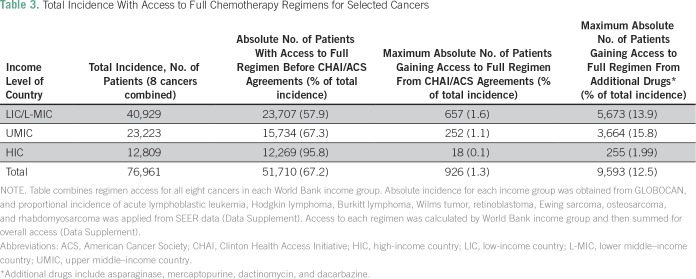
Total Incidence With Access to Full Chemotherapy Regimens for Selected Cancers

We leveraged our survey’s availability data to identify a combination of drugs that could more dramatically reduce the coverage gap (Fig 3). This sensitivity analysis indicated that adding a four-drug package of asparaginase, mercaptopurine, dactinomycin, and dacarbazine to the CHAI/ACS list would increase full regimen access by a factor of 10 (12.5%) worldwide compared with the CHAI/ACS agreements alone. This projects to 926 patients affected by the CHAI/ACS agreements compared with 9,600 patients gaining full regimen access if the four drugs we identified were added to the CHAI/ACS agreements list (Table 3).

## DISCUSSION

We have comprehensively assessed the practical availability of vital chemotherapeutics globally for children with cancer and have defined disparities that may inform the implementation of cancer control plans that are sensitive to the pediatric population. We used two different but complementary approaches to quantify access. First, we applied a proportional scale, the access score, to broadly map the availability of EMLc cytotoxic and adjuvant therapies. Second, we developed a novel and more clinically relevant disease-specific approach by examining access on the basis of packages of chemotherapy agents required to cure common pediatric cancers. Although the access score provides a gross appreciation of overall EMLc drug access that can be easily compared across regions and income groups, our package-based approach yields more actionable, policy-oriented outcomes data to guide interventions.

The access score results build on the work of Barr and Robertson^5^ that showed the number of drugs on national formularies correlated with development indicators such as World Bank income group. Because the median year of the national drug lists that they referenced was 2010, it is difficult to attempt direct comparisons with our results. Since 2010, there have been four revisions to the EMLc, such that there are nine drugs that appear exclusively on either the 2010 or 2017 list.^14,20^ Despite these important caveats, we note that Barr and Robertson^5^ found a median proportion of essential chemotherapeutics listed in L-MIC formularies of 77.8% (14 of 18 drugs), with is higher than the median in L-MICs in our study (69.5%; 15.3 of 22 drugs). In addition, they found a median proportion of essential chemotherapy drugs among LICs of 38.9% (seven of 18 drugs), which was lower than our results (56.8%; 12.5 of 22 drugs).

In contrast with using national drug formularies, our treatment center–based survey approach accounts for both practical drug stock availability and in-country variability. Regarding the former, a follow-up of the work by Barr and Robertson^5^ reviewing adult formularies discussed the need for complementary in-country accessibility data, noting that market supply barriers can make formulary medications unobtainable.^21^ Indeed, low supply of chemotherapy as a result of global shortages is a problem faced in HICs and LMICs.^22^ Our data identified a frequent role for nongovernment actors in the procurement of chemotherapeutic agents in LICs/L-MICs, which may result in availability of unlisted drugs in countries with spare national formularies. Considering variability, a survey approach allows multiple responses per country, thus accounting for differences between centers that would be otherwise obscured by a single national list. Indeed, heterogeneity in other aspects of childhood cancer care has been found in LMICs and associated with measures of domestic inequality.^8,23^ Thus, we operated from the paradigm of in-country variability in access as the norm, although future studies may attempt to better characterize heterogeneity of centers within countries.

Access scores were lower in LICs/L-MICs compared with UMICs or HICs. Analysis of countries with access scores that outperformed their peers may provide a signal for additional case studies and policy interventions. Among L-MICs, optimal access scores in Sri Lanka and Indonesia might be related to marked investments in tenets of universal health coverage, whereas those in Central American countries and Tanzania could reflect successful twinning efforts with HIC centers that have fostered local capacity building.^24-26^

Whereas the access score was conceived as a gross measure of availability, the regimen-based analysis encourages patient centeredness in drug procurement and policy making. We believe it can serve as a conceptual framework to help investigators incorporate access as a key trade-off parameter (with survival benefit and toxicity) when designing future adapted regimens and treatment guidelines for low-income settings. The disease-specific sensitivity analyses underscore that pediatric cancers require distinct consideration in future public-private negotiations. We identified a 10-fold increase in access by the simple addition of four common cytotoxic medications to the existing CHAI/ACS drug list. Although the current agreements remain limited to six LMICs in sub-Saharan Africa, the CHAI/ACS efforts represents a watershed moment for increasing global access to cytotoxic cancer therapy, and the potential impact of this model initiative on the entire field of global oncology cannot be overstated. Our results should not be viewed as a critique but rather a prescriptive blueprint to help guide future negotiations so that more children may also benefit.

There are several limitations that should be considered in interpreting this study. Our study was limited to respondents who could answer in English. Because Cure4Kids is an English-language Web site, we felt its users would have basic English proficiency. Other sample pools would have improved the study’s validity. Nearly all respondents were physicians. Surveying pharmacists may have reduced bias but practically would have been difficult given that such positions are less common in LMICs. An inventory analysis would have eliminated response bias, but to our knowledge, there is no central institution collecting international inventory data for pediatric cancer.

The overall response rate decreased within the norms previously described regarding e-mail response rates in medical research, as well as previous published studies using Cure4Kids.^8-10,27^ Still, the limited sample may misrepresent the true access to chemotherapy in individual countries. The number of responses per country varied greatly (range, one to 60 responses), with nearly half of countries (46 of 94 countries) yielding two or fewer respondents. This may be suitable for smaller LMICs, where there are few pediatric cancer centers, but not larger countries where substantial regional heterogeneity exists. A higher response rate would have increased the representativeness of the sample and potentially improved the accuracy and precision of our results. That said, using the Cure4Kids register is an established approach in the absence of a global registry, and this study provides a methodologic framework for future investigations to improve the accuracy and precision with larger samples. We believe that this limitation is mitigated somewhat in the regimen-based analyses that used World Bank income groups (140 or more analyses for each group) as the comparator.

Finally, our approach used center-based data to make population-based estimates. We recognize that access is multidimensional; affordability, acceptability, and quality control intersect with availability to compound the associations between income level and drug access that we found.^6^ As recently seen in Brazil, the procurement of cheaper generic alternatives may result in serious safety risks to patients in the absence of comprehensive safety data.^28^ We intend the regimen-based sensitivity analysis to be hypothesis generating and guide countries’ situation analyses of access.

In estimating regimen-based access and the relative impact of the CHAI/ACS agreements, we were forced to make decisions about which regimens and what epidemiologic sources to use. Although multiple protocols exist in resource-limited settings, we opted to preferentially use those developed by the WHO-sanctioned Expert Committee and endorsed by the International Society of Pediatric Oncology. We saw these regimens as consensus guidelines and representative for the broadest population. To maximize their potential effect, our estimations of the impact of the CHAI/ACS agreements assumed full availability of the included drugs worldwide; however, the agreements currently only apply to the initial six-country cohort. Finally, this study applied SEER proportional incidence data to determine the relative frequencies of cancers globally, despite known incidence differences geographically.^29^ The lack of modeled global pediatric cancer incidence data made our approach the most feasible option for a sensitivity analysis by World Bank income group.^30^

To summarize, this study confirms that there is a wide gap in practical availability of essential pediatric chemotherapy between HICs, UMICs, and LIC/L-MICs, leading to tens of thousands of potentially preventable deaths every year. Using a disease-specific approach with multidrug packages, we described a mechanism to inform future public-private agreements to improve benefit for children. These data should inform resource-adapted chemotherapy regimens so that access is also considered within guidelines for cancer treatment in LMICs.
